# CMR measures of left and right ventricular systolic performance and mortality: a factor analysis

**DOI:** 10.1186/1532-429X-18-S1-P144

**Published:** 2016-01-27

**Authors:** Thomas H Hauser, Evan Appelbaum, Eli V Gelfand, Loryn Feinberg, TD O'Halloran, Kraig V Kissinger, Warren J Manning

**Affiliations:** 1 Cardiology, BIDMC, Boston, MA USA; 2 grid.416843.c000000040382382XMount Auburn Hospital, Cambridge, MA USA

## Background

Left ventricular (LV) and right ventricular (RV) systolic performance, typically measured as the ejection fraction (EF), is strongly correlated with mortality. However, CMR provides multiple measures that may also reflect systolic performance including LV volumes and cardiac outflow. We examined the predictive relationship of multiple potential measures of cardiac systolic performance with mortality in a large CMR clinical database.

## Methods

A consecutive series of 2307 study subjects who underwent clinical CMR from 2002 to 2012 for which complete data were available were included in the study. CMR was performed using a 1.5T system (Philips Achieva). LV functional and flow images were acquired and analyzed according to standard clinical protocols. Vital status was determined using the Social Security Administration Death Master File. Proportional hazards regression was performed to assess the relationship between imaging measures and mortality. Factor analysis was performed to identify groups of highly correlated imaging measures.

## Results

The characteristics of the study cohort are shown in the Table. During a mean follow-up of 3.1 years, there were 134 deaths. Age and LV end systolic volume (ESV) were associated with increased mortality while cardiac output (CO), cardiac index (CI), LVEF, effective (eff) LVEF, LV stroke volume (SV), RV end diasatolic volume (RVEDV), RVEDV index, RVSV, and RVEF were inversely associated with mortality. Multivariate regression failed due to multicolinearity. Factor analysis identified 2 independent factors. The Figure shows the loading pattern. RVEF, LVEF, effLVEF, and the inverse (inv) of LVESV loaded on the first factor, termed the cardiac ejection factor. CO, CI, LVSV and RVSV loaded on the second factor, termed the cardiac outflow factor. RVEDV and RVEDV indx also loaded on the outflow factor. Both factors had strong, independent inverse associations with mortality (Table 1).

**Table 1:** Characteristics of the study cohort and association with mortality.

Variable	Value	Hazard Ratio	P
Age,y*	51 ± 16	1.67(1.46-1.90)	<0.001
Male	1316(57%)	0.91(0.65-1.28)	0.593
CO,L/min*	5.4 ± 1.5	0.84-0.75-0.95)	0.004
CI,L/min/m2*	2.8 ± 0.7	0.67(0.52-0.87)	0.002
LVEDV,ml*	170 ± 58	1.00(0.97-1.03)	0.836
LVEDV index, ml/m2*	0.87 ± 26	1.02(0.95-1.08)	0.627
LVESV,ml*	74 ± 48	1.04(1.01-1.07)	0.005
LVSV, ml*	97 ± 28	0.87(0.81-0.93)	<0.001
LVEF,%*	59 ± 12	0.76(0.67-0.86)	<0.001
Effective LVEF,%*	51 ± 12	0.71(0.63-0.79)	<0.001
RVEDV,ml*	161 ± 53	0.96(0.92-1.00)	0.027
RVEDV index,ml/m2*	82 ± 24	0.90(0.82-0.98)	0.018
RVESV,ml*	73 ± 36	0.99(0.94-1.04)	0.745
RVSV,ml*	89 ± 27	0.86(0.80-0.93)	<0.001
RVEF,%*	56 ± 9	0.84(0.70-1.00)	0.049
Cardiac ejection factor	NA	0.74(0.65-0.85)	<0.001
Cardiac outflow factor	NA	0.67(0.55-0.81)	<0.001

*Hazard ratio shown for a 10 unit change.

Abbreviations as in the text.

**Figure 1:**
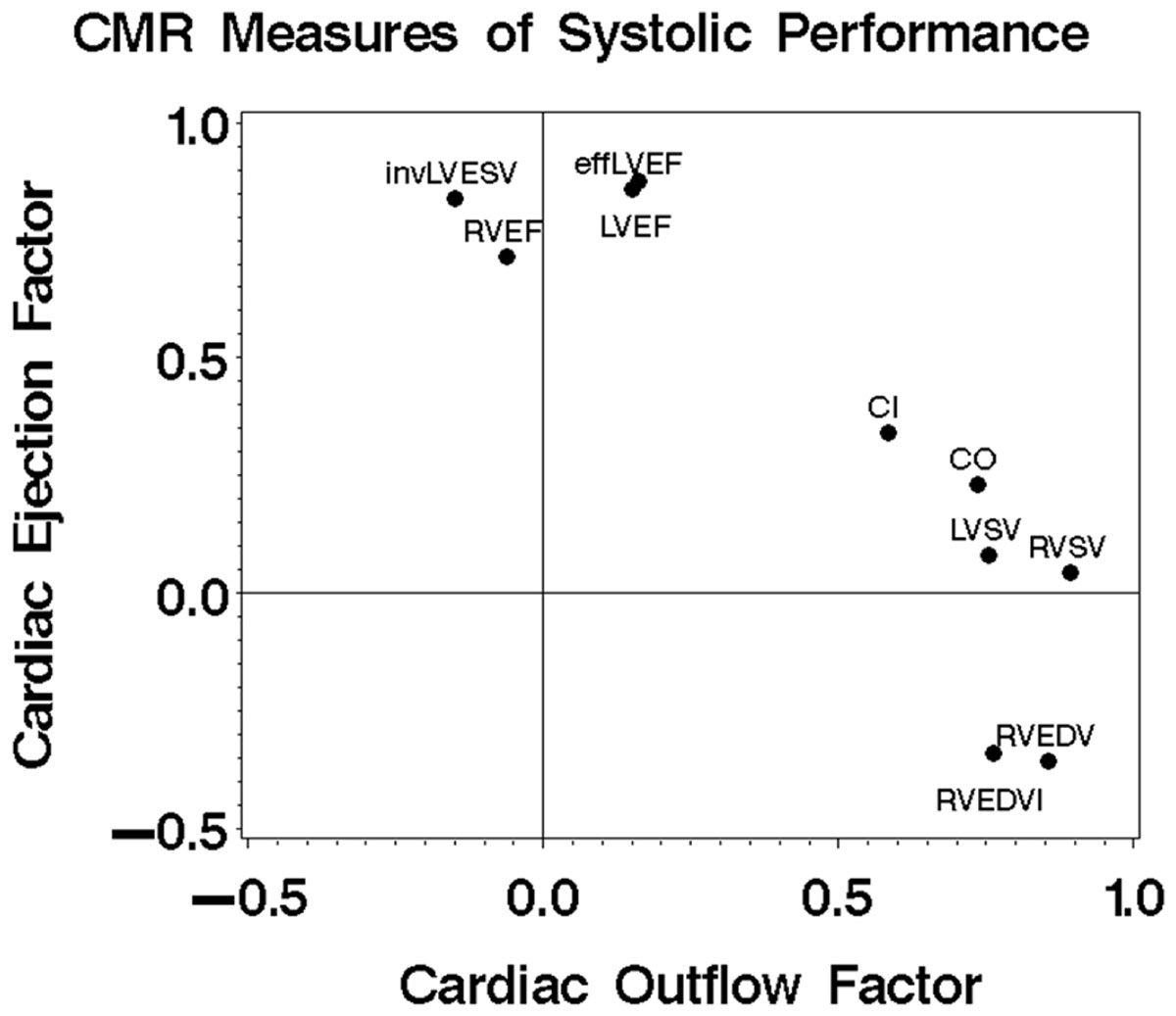
**Loading pattern for CMR measures of left and right ventricular systolic performance on the cardiac ejection factor and the cardiac outlfow factor**.

## Conclusions

Multiple CMR measures of cardiac systolic performance correlate with mortality. Two independent factors of systolic performance, cardiac ejection and cardiac outflow, had strong inverse associations with mortality and may represent distinct physiologic measures of LV systolic performance.

